# Supplementary material for: Polydopamine-Coated Co3O4 Nanoparticles as an Efficient Catalase Mimic for Fluorescent Detection of Sulfide Ion
Source: Biosensors (Basel). 2022 Nov 19;12(11):1047. doi: 10.3390/bios12111047 (PMC9688345; doi:10.3390/bios12111047)
Supplement: Supplementary file 1 [file biosensors-12-01047-s001.zip › biosensors-2012817-supplementary.pdf]

# Supplementary Materials

# Polydopamine-Coated $Co_3O_4$ Nanoparticles as an Efficient Catalase Mimic for Fluorescent Detection of Sulfide Ion

**Trung Hieu Vu, Phuong Thy Nguyen, and Moon Il Kim ***

Department of BioNano Technology, Gachon University, 1342 Seongnamdae-ro, Sujeong-gu, Seongnam 13120, Republic of Korea
* Correspondence: moonil@gachon.ac.kr; Tel.: +82-31-750-8563

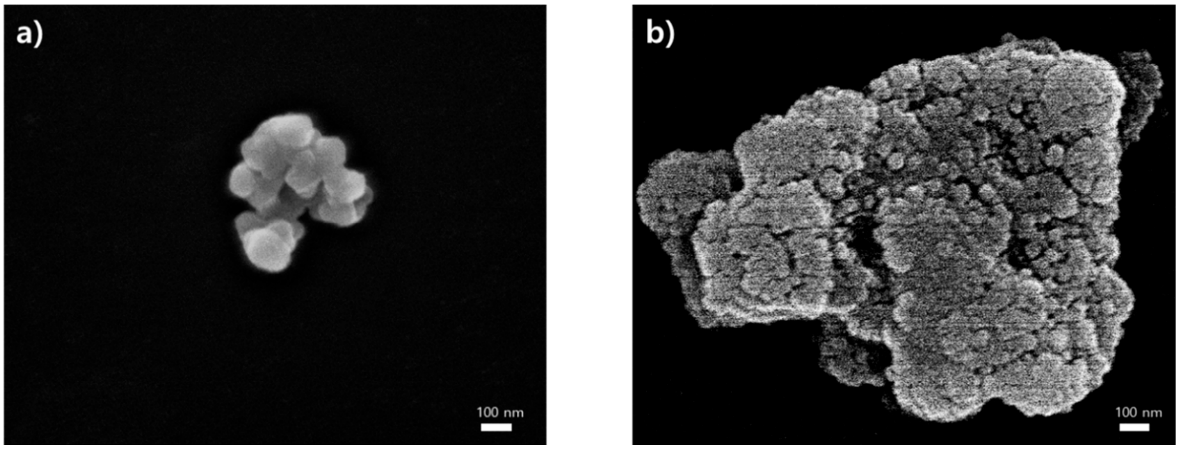


**Figure S1.** SEM images of a) bare $Co_3O_4$ NPs and b) PDA@$Co_3O_4$ NPs. Scale bar: 100 nm.

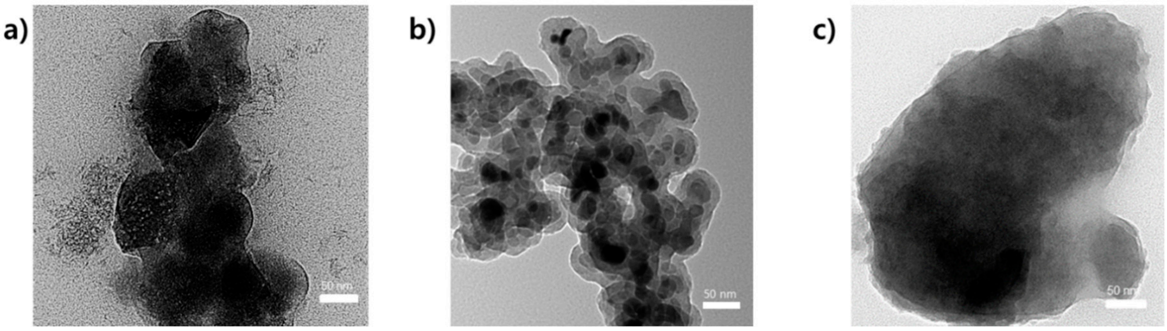


**Figure S2.** TEM images of a) 0.5-PDA@$Co_3O_4$ NPs, b) 1-PDA@$Co_3O_4$ NPs, and c) 2-PDA@$Co_3O_4$ NPs. Scale bar: 50 nm.

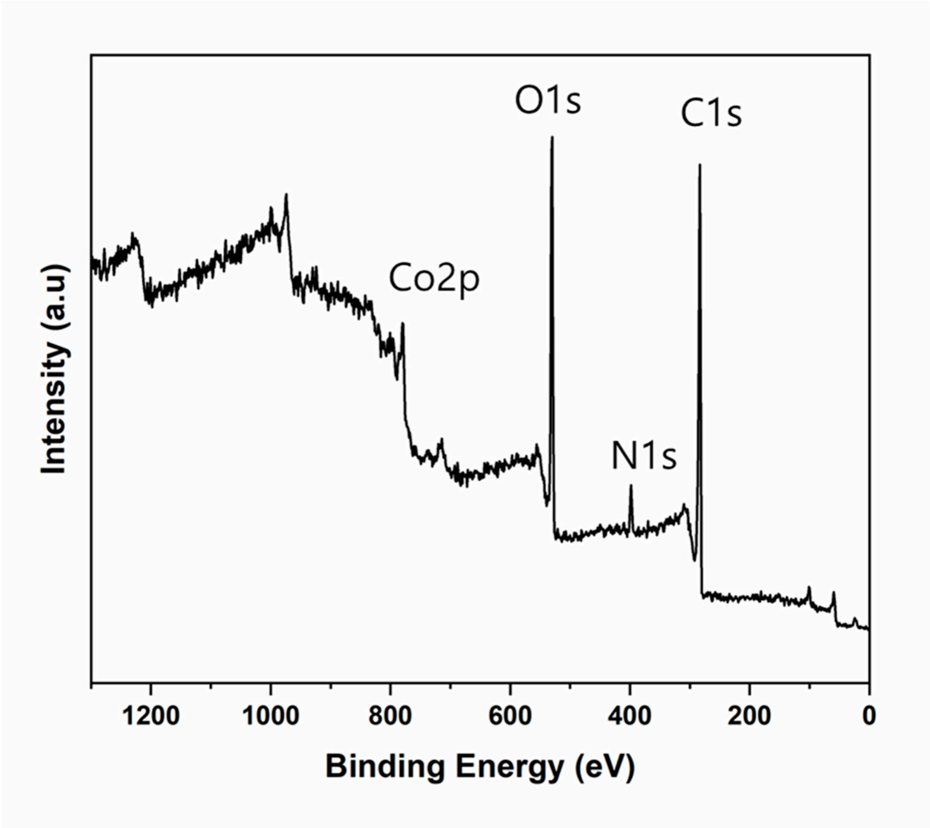


**Figure S3.** XPS spectra of PDA@$Co_3O_4$ NPs.

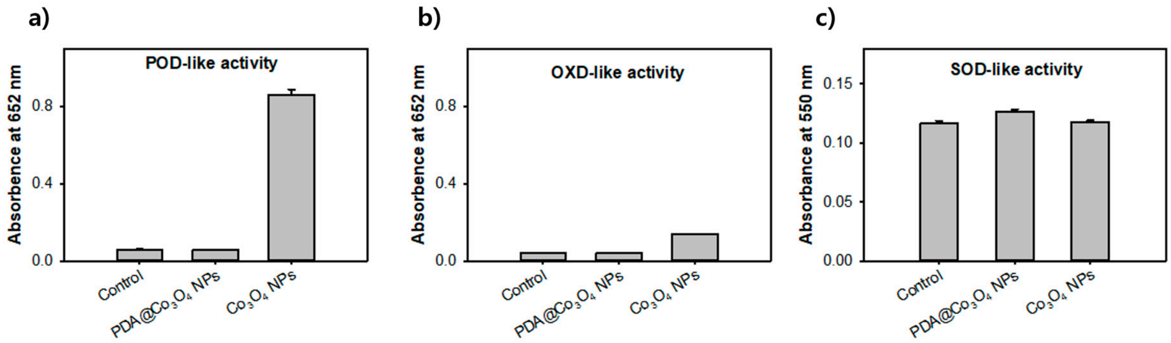


**Figure S4**. Evaluations for the other oxidoreductase-like activities of PDA@$Co_3O_4$ NPs and bare $Co_3O_4$ NPs. a) POD-, b) OXD- and c) SOD-like activities.

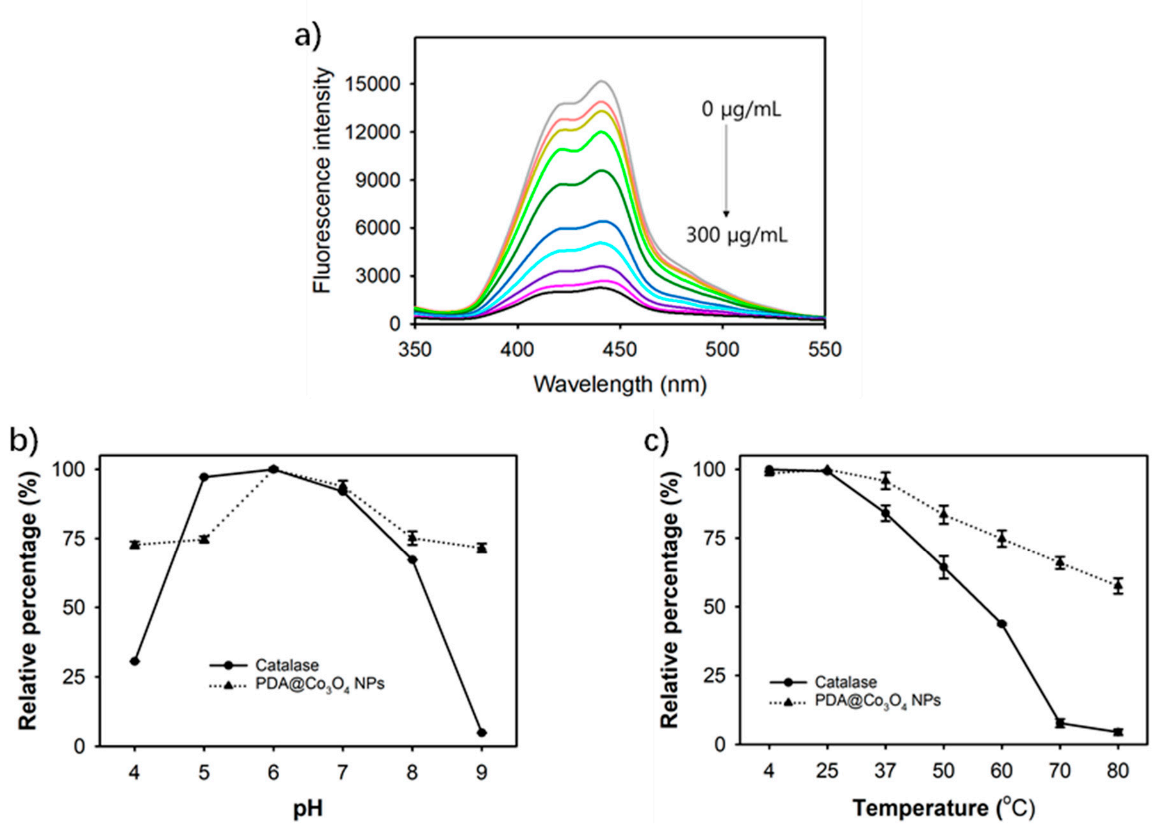


**Figure S5**. Effects of a) concentrations of PDA@$Co_3O_4$ NPs, b) pH, and c) temperature on the catalase-like activity of PDA@$Co_3O_4$ NPs. Effects of pH and temperature on the activity of natural catalase were compared.

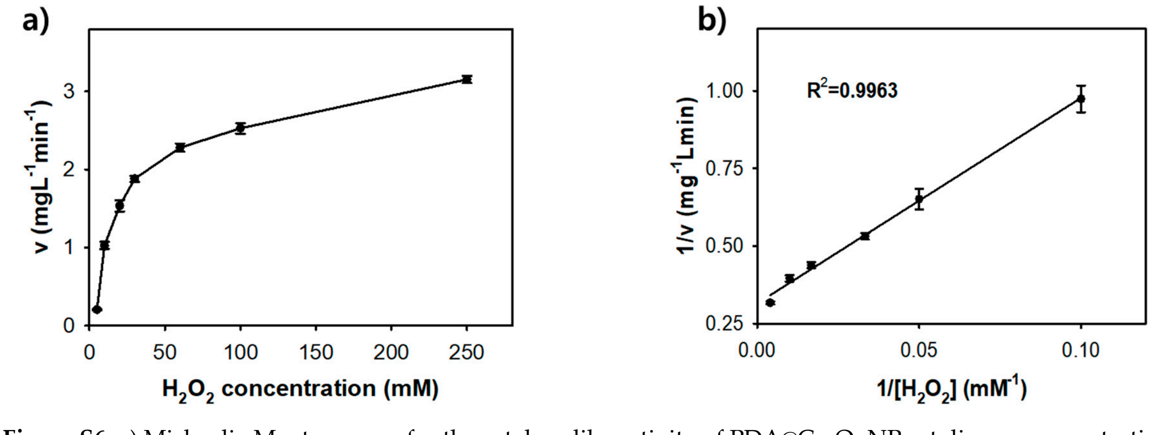


**Figure S6.** a) Michaelis-Menten curve for the catalase-like activity of PDA@$Co_3O_4$ NPs at diverse concentrations of $H_2O_2$ and b) their corresponding Lineweaver-Burk plots (n=3).

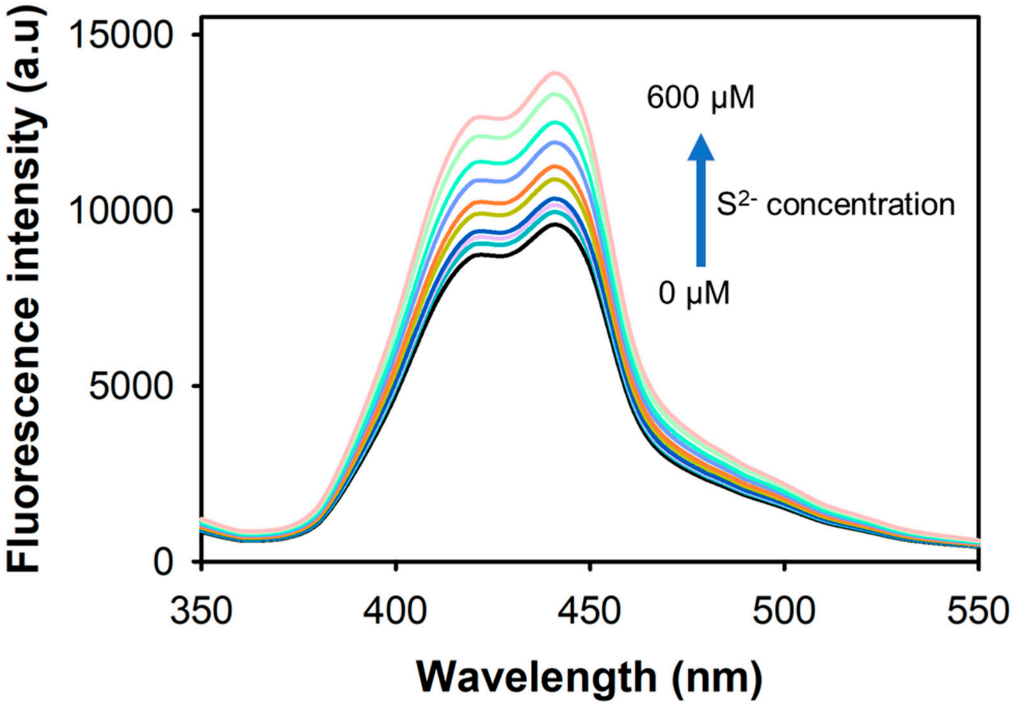


**Figure S7.** Fluorescence spectra of PDA@$Co_3O_4$ NPs-based biosensor toward diverse concentrations of $S^{2-}$.

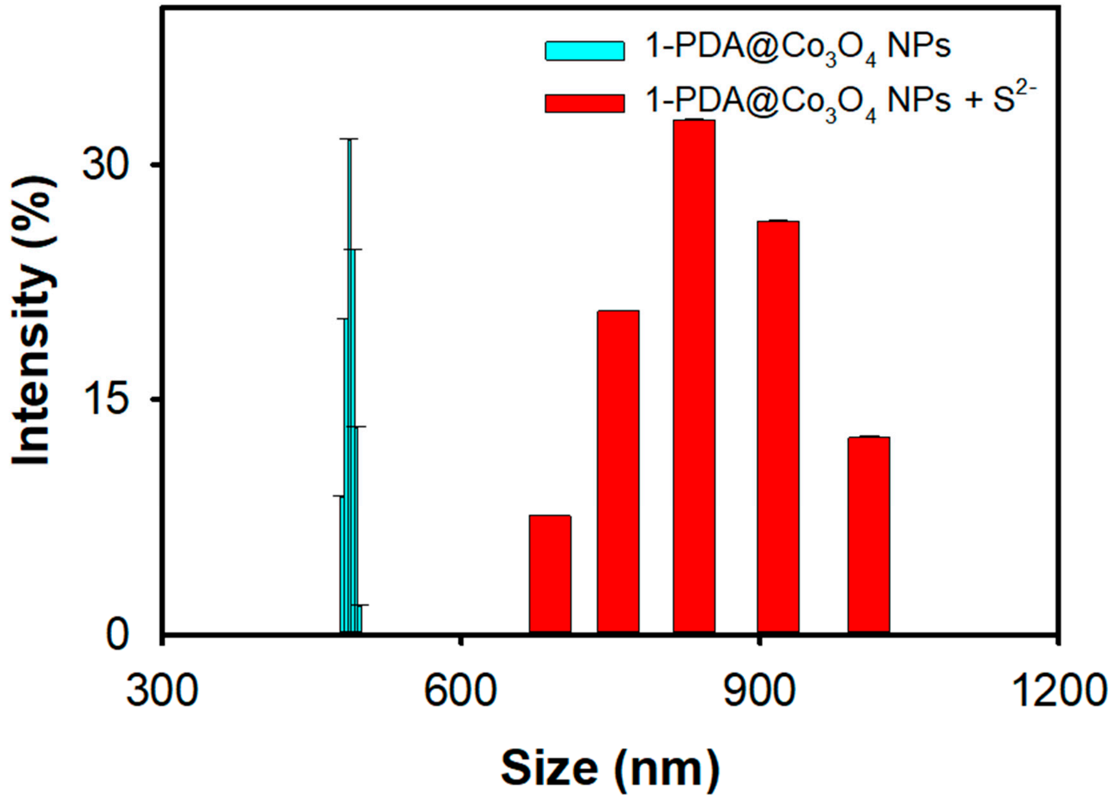


**Figure S8.** Particle size distributions of PDA@$Co_3O_4$ NPs in the absence and presence of $S^{2-}$ (1 mM).

**Table S1.** Elemental composition ratio of PDA@$Co_3O_4$ NPs.

| Element | Weight % | Atomic % |
|---|---|---|
| C | 36.68 | 57.90 |
| N | 3.39 | 4.59 |
| O | 21.10 | 25.01 |
| Co | 38.83 | 12.50 |

**Table S2.** Comparison of the kinetic parameters of catalase-like PDA-$Co_3O_4$ NPs with those of natural catalase and previously reported $Co_3O_4$-based nanozymes.

| Catalyst | $K_m$ (mM) | $V_{max}$ ($10^{-5}$ M/s) | References |
|---|---|---|---|
| $Co_3O_4$ NPs | 34.3 | 1.12 | [1] |
| Catalase | 54.3 | 1.62 | |
| $Co_3O_4$ nanoplates | 24.7 | 0.24 | [2] |
| $Co_3O_4$ nanorods | 4.82 | 0.19 | |
| $Co_3O_4$ nanocubes | 63.9 | 0.12 | |
| PDA@$Co_3O_4$ NPs | 22.1 | 0.67 | **This work** |

## References

[1] Mu, J.; Zhang, L.; Zhao, M.; Wang, Y. Co3O4 nanoparticles as an efficient catalase mimic: Properties, mechanism and its electrocatalytic sensing application for hydrogen peroxide, *J. Mol. Catal. A-Chem*. **2013**, *378*, 30-7.

[2] Mu, J.; Zhang, L.; Zhao, M.; Wang, Y. Catalase mimic property of Co3O4 nanomaterials with different morphology and its application as a calcium sensor, *ACS Appl. Mater. Interfaces*. **2014**, *6*, 7090-8.
